# Long-Haul Northeast Travel Disrupts Sleep and Induces Perceived Fatigue in Endurance Athletes

**DOI:** 10.3389/fphys.2018.01826

**Published:** 2018-12-20

**Authors:** Christopher J. Stevens, Heidi R. Thornton, Peter M. Fowler, Christopher Esh, Lee Taylor

**Affiliations:** ^1^School of Health and Human Sciences, Southern Cross University, Coffs Harbour, NSW, Australia; ^2^Centre for Athlete Development, Experience and Performance, Southern Cross University, Coffs Harbour, NSW, Australia; ^3^Newcastle Knights Rugby League Club, Newcastle, NSW, Australia; ^4^La Trobe Sport and Exercise Medicine Research Centre, La Trobe University, Melbourne, VIC, Australia; ^5^School of Exercise and Nutrition Sciences, Queensland University of Technology, Brisbane, QLD, Australia; ^6^Athlete Health and Performance Research Centre, ASPETAR Qatar Orthopaedic and Sports Medicine Hospital, Doha, Qatar; ^7^School of Sport, Exercise and Health Sciences, Loughborough University, Loughborough, United Kingdom

**Keywords:** air travel, hot environment, sleep, immunity, illness, fatigue, salivary IgA, salivary cortisol

## Abstract

**Introduction:** Long-haul transmeridian travel is known to cause disruptions to sleep and immune status, which may increase the risk of illness.

**Aim:** This study aimed to determine the effects of long-haul northeast travel for competition on sleep, illness and preparedness in endurance athletes.

**Methods:** Twelve trained (13.8 ± 3.2 training h/week) masters (age: 48 ± 14 years) triathletes were monitored for sleep (quantity via actigraphy and quality via self-report), mucosal immunity (salivary immunoglobulin-A) and stress (salivary cortisol) as well as self-reported illness, fatigue, recovery and preparedness. Baseline measures were recorded for 2 weeks prior to travel for all variables except for the saliva samples, which were collected on three separate days upon waking. Participants completed normal training during the baseline period. Measures were subsequently recorded before, during and after long-haul northeast travel from the Australian winter to the Hawaiian summer, and in the lead up to an Ironman 70.3 triathlon.

**Results:** All comparisons are to baseline. There was a *most likely* decrease in sleep duration on the over-night flight (-4.8 ± 1.2 h; effect size; ±90% confidence limits = 3.06; ±1.26) and a *very likely* increase in sleep duration on the first night after arrival (0.7 ± 1.0 h; 1.15; ±0.92). After this time, sleep duration returned to baseline for several days until it was *very likely* decreased on the night prior to competition (-1.2 ± 1.0 h; 1.18; ±0.93). Nap duration was *likely* increased on the first day after arrival (36 ± 65 min; 3.90; ±3.70). There was also a *likely* increase in self-reported fatigue upon waking after the first night in the new destination (1.1 ± 1.6 AU; 0.54; ±0.41) and there were three athletes (25%) who developed symptoms of illness 3–5 days after arrival. There were no changes in sleep quality or mucosal measures across study.

**Discussion:** Long-haul northeast travel from a cool to a hot environment had substantial influences on sleep and self-reported fatigue, but these alterations had returned to pre-departure baseline 48 h after arrival. Endurance athletes undertaking similar journeys may benefit from optimizing sleep hygiene, especially on the first 2 days after arrival, or until sleep duration and fatigue levels return to normal.

## Introduction

Insufficient sleep and/or illness can disrupt an athlete’s training, competition and performance-recovery (Pyne et al., 2005; Rae et al., 2017). Athletes are susceptible to reductions in sleep duration and quality during training and proximal to competition, especially the night prior-to competition (Juliff et al., 2015; Roberts et al., 2018), albeit subject to high inter-and intra-individual variability (Nedelec et al., 2018). Athletes often relocate from various global locations (e.g., home training base, pre-competition or holding-camp) to compete at major competitions, meaning many athletes will relocate several times across numerous continents before a competition. Sleep loss has consistently been reported during long-haul travel in economy class (Fowler et al., 2015, 2017), likely due to the cabin conditions, including the uncomfortable sleeping position (Roach et al., 2018). Light, noise and the timing of meals and stopovers may also contribute to sleep disruption on a long-haul flight. Airliner cabin conditions can increase the risk of illness, particularly drying of the respiratory epithelium due to the low humidity, close contact with fellow infectious travelers and exposure to their re-circulated air (Schwellnus M. P. et al., 2012b; Svendsen et al., 2016). Moreover, athletes could be at greater risk of travel induced illness compared to the general population, since prolonged exercise and intensified training are known to suppress mucosal immunity, increasing the risk of upper respiratory tract infections (URTI) (Walsh et al., 2011). Moreover, insufficient sleep itself increases susceptibility to respiratory infections and airborne viruses (Prather et al., 2015; Prather and Leung, 2016).

Long-haul transmeridian air-travel can elicit jet-lag symptomology, predominately due to misalignment between body clock time (as indicated by the circadian rhythm in body temperature or melatonin) and local time at the new location (Reilly et al., 2005; Forbes-Robertson et al., 2012). Jet-lag mediated sleep disruption is not uncommon, with delayed sleep onset and early awakening common after eastward and westward travel, respectively (Thun et al., 2015; Fowler et al., 2017). Such responses can temporarily reduce facets of performance (Fullagar et al., 2015), with emerging evidence suggesting that direction of travel (e.g., eastward travel eliciting a greater detrimental performance effect than westward) is important (Fowler et al., 2017). Other detrimental physiological and perceptual responses are also seen (Fullagar et al., 2015). While several studies have characterized sleep responses to east and westward travel (Thun et al., 2015; Fowler et al., 2017), there is a lack of research on long-haul travel that causes little disruptions to the sleep-wake cycle (i.e., little jet-lag), where fatigue from the travel itself can be studied without influences from large shifts in time-zone.

Factors associated with the destination may also have a negative influence on sleep and illness risk upon arrival. The environmental conditions, food, and exposure to different pathogens upon arrival were purported as reasons for the twofold to threefold increase in the incidence of all illness in professional rugby union players following international travel (Schwellnus M. et al., 2012a). A large climatic contrast between the place of departure and destination is also suggested to be a risk factor for illness (Schwellnus M. et al., 2012a) and perhaps sleep disruption. For example, given the circadian influence of a reduction in body temperature on sleep onset (Wyatt et al., 1999), exposure to and/or exercise in the heat, particularly in the afternoon/evening, may negatively affect sleep (Buguet, 2007). Lastly, athletes training/competing in the heat may be at increased risk of heat-related illness (Racinais et al., 2015), especially if they are coming from a cooler climate and have not been able to completely heat acclimatize (Heathcote et al., 2019). Hence, athletes traveling from a cool to a hot environment may be at increased risk of sleep disruption, lowered immunity and illness.

Therefore, the aim of the current study was to determine the effects of long-haul northeast travel from a cool (Winter of New South Wales, Australia) to a hot environment (Summer of Hawaii, United States) for competition on sleep, mucosal immunity and stress, as well as illness, fatigue, recovery and preparedness in endurance athletes. We hypothesize that the travel will; (i) decrease sleep duration on the day of the flight; (ii) decrease salivary IgA (sIgA) concentration on arrival; and (iii) negatively influence self-reported fatigue, recovery and/or preparedness on arrival, compared to baseline.

## Materials and Methods

### Participants

Twelve masters level triathletes (age: 48 ± 14 years, height: 172 ± 11 cm, body mass: 72 ± 11 kg) volunteered to participate in the study. The athletes had a mean weekly training duration of 13.8 ± 3.2 h (range = 10.4–20.2 h) across the first 3 weeks of May 2017 (i.e., the last month before competition but prior to commencing a taper on the day of departure). The sample included both ‘trained’ and ‘well-trained’ athletes according to published guidelines (De Pauw et al., 2013). Training was completed on the mid-north coast of Australia, predominantly during early mornings of the autumn season (10–15°C). Hence, the athletes were not seasonally acclimatized, however, they did perform three to five training sessions in additional clothing (Stevens et al., 2017) within 4 weeks of the race. The protocol was approved by the Southern Cross University Human Research Ethics Committee. All subjects gave written informed consent in accordance with the Declaration of Helsinki.

### Design

**FIGURE 1:**
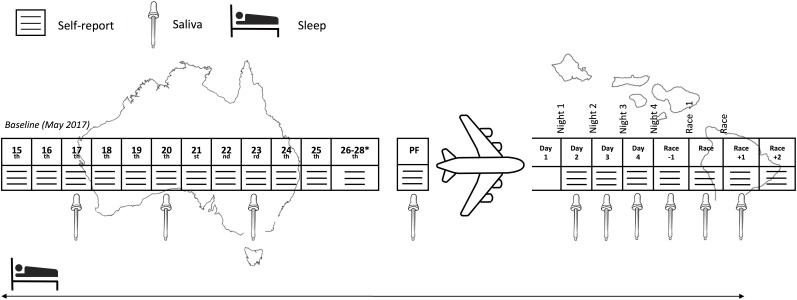
Experimental schematic of the measures across the study period. PF, pre-flight. ^∗^Inclusion of these days in the baseline period was subject to the participant’s departure date.

**FIGURE 2:**
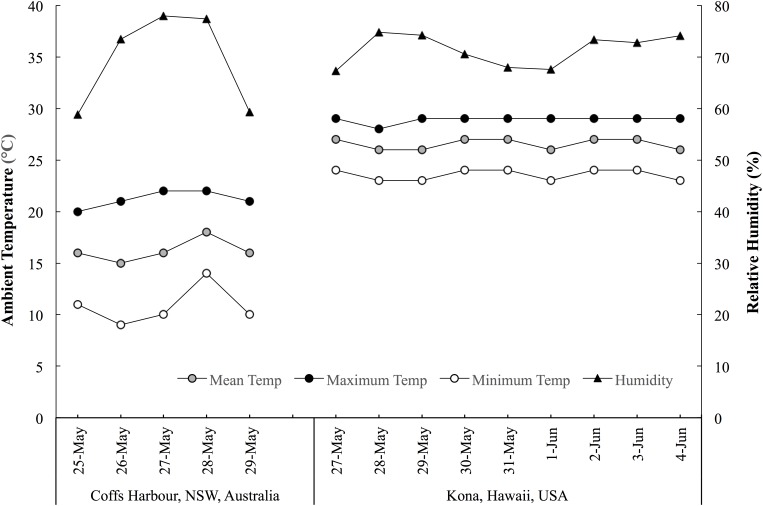
The mean, maximum and minimum ambient temperatures, and mean daytime humidity experienced by the athletes in the days prior to and throughout the travel period. Data acquired from www.wunderground.com/weather.

This prospective cohort study monitored sleep (quality and quantity), mucosal markers of immunity (sIgA) and stress (salivary cortisol [sCort]) as well as self-reported symptoms of illness, fatigue, recovery and preparedness before and after long-haul transmeridian travel from the Australian Winter to the Hawaiian Summer in preparation for the 2017 Hawaii Ironman 70.3 triathlon. An experimental schematic of the timeline of the measures is illustrated in Figure 1. Figure 2 illustrates the mean, maximum and minimum ambient temperatures, as well as the mean daily humidity experienced by the athletes, in the days prior to travel and after arrival in the new destination.

### Travel

The travel involved 22.6 ± 2.4 h of door–door travel time including 11.5 h of flight time. All athletes traveled in economy class for all flights. The journey involved;

(1) Road transfer from individual homes (15:00–17:00 AEST) to Coffs Harbour airport, Australia (0.1–0.5 h)
(2) Flight to Sydney, Australia (1.25 h)
(3) Flight to Honolulu, United States (9.5 h)
(4) Flight to Kona, United States (0.75 h)
(5) Road transfer from Kona airport to accommodation in Waikoloa, United States (0.75 h; arriving between 15:30 and 17:00 HST).

Stopovers in Sydney and Honolulu ranged from 2 to 5 h depending on the airline, but all athletes experienced the same flight duration (and hence very similar routines on the flights themselves in terms of meals and light exposure). Eight of the athletes departed on the 29th, three departed on the 28th and one departed on the 27th of May, 2017, arriving at the accommodation on the same day that they departed due to the shift in time zone (-22 h).

### Measures

#### Sleep

Sleep was monitored using self-report diaries and wrist activity monitors (wActiSleep+, Actigraph, FL, United States) for 3 weeks (15th May to the 4th June 2017). According to previously described methods, data from the sleep diaries and activity monitors were used to determine when participants were awake and asleep (Sargent et al., 2016). All time was scored as wake unless: (i) the sleep diary indicated that the participant was lying down attempting to sleep and (ii) the activity counts from the monitor were sufficiently low to indicate that the participant was immobile (i.e., where the weighted activity count for an epoch fell below the defined threshold). When these two conditions were satisfied simultaneously, time was scored as sleep. This scoring process was conducted using the corresponding software (Actilife, version 6.13.3, Actigraph, FL, United States) and Cole-Kripke algorithm, which has been validated for use in adults (Cole et al., 1992). The following variables were derived from the sleep diary and activity monitor data; sleep duration (h:min): the amount of time spent in bed asleep and sleep efficiency (%): sleep duration expressed as a percentage of time in bed. Participants also self-reported nap duration (min; there was no minimum) and sleep quality (where 1 = very poor, 2 = poor, 3 = fair, 4 = good, 5 = very good). All sleep on the flight(s) was scored via actigraphy as ‘night time sleep’ i.e., participants were instructed to exclude this from their self-reported nap duration.

#### Mucosal

The sIgA and sCort were determined from saliva samples analyzed with an IPRO cube reader (IPRO Interactive, Wallingford, United Kingdom), that has been deemed valid (*r* = 0.93) and reliable (ICC = 0.89, CV = 9.4%) for the measurement of sIgA (Coad et al., 2015) and valid (*r* = 0.52) and reliable (*r* = 0.69) for measurement of sCort (Fisher et al., 2015). All samples were collected upon waking within a 1-h window for each individual (with the exception of race day, where wake time was 3:30 AM) and prior to any exercise, food or fluid ingestion. An oral swab was placed on the top of the tongue, and when an adequate volume of saliva (0.5 mL) was collected, the volume-indicator turned blue. Swabs were then placed into a buffer solution provided by the manufacturers, and two drops of the buffer-saliva mixture were placed on the dual-cassette test strip. Following a 10-min incubation period, the test strip was placed in the IPRO to determine raw concentrations of sIgA (μg^.^mL^-1^), and sCort (ng^.^mL^-1^). As per Figure 1, saliva samples were collected on three occasions at baseline in the week prior to travel, and another sample was collected on the day of the travel. Further, samples were collected on the day after arrival and then on a daily basis until the day following the event. The mucosal data excluded one participant who reported illness in the days prior to the flight, which persisted throughout the flight and for 2 days after arrival. A further three participants were also removed due to inadequate sample preparation/technical problems.

#### Self-Report

The athletes self-reported several measures on a daily basis upon waking throughout the study period. This included symptoms of illness via the athlete illness questionnaire (Matthews et al., 2010), training load via session-rating of perceived exertion (Foster et al., 2001) as well as measures of fatigue (“rate your fatigue over the last 24 h”; 0 = no fatigue, 10 = as bad as you can imagine); recovery (“how well recovered are you?”; 0 = very poorly, 10 = very well); physical preparedness (“how physically ready are you for strenuous exercise?”; 0 = not ready at all, 10 = totally ready), and; mental preparedness (“how mentally ready are you for strenuous exercise?”; 0 = not ready at all, 10 = totally ready).

### Data Analysis

For the purposes of analysis, the following periods/days were defined as per Figure 1; *Baseline:* Two-week period immediately prior to the travel, where athletes lived in their usual home environment and continued with routine training; *Pre-flight:* The day/night prior to travel; *Flight:* The data recorded during the international flight; *Days/Nights 1 to 4:* First 4 days/nights after arrival; *Race -1:* Day/night prior to the race; *Race:* Day/night of the race; *Race +1*: Day following the race; *Race +2*: Two days following the race. For the mucosal markers, both the raw data and the change (delta; Δ) from the individual’s baseline were determined.

### Statistical Analysis

Measurements are presented as mean ± standard deviation (SD) and were analyzed using a non-clinical magnitude-based inference approach (Hopkins et al., 2009). The data were log-transformed (i.e., 100 × natural log) and the magnitudes of the changes between trials were expressed as standardized differences (effect sizes; ES) with 90% confidence limits (CL). The criteria used for interpreting the magnitude of the ES were: ≤0.2 (trivial), >0.2 (small), >0.6 (moderate), >1.2 (large) and >2.0 (very large) as described previously (Hopkins et al., 2009). If the 90% CL overlapped positive and negative trivial ES values then the effect was deemed unclear. The quantitative chances of differences being substantial were assessed qualitatively as follows: 75–95% (*likely*); 95–99% (*very likely*); >99% (*most likely*) as described previously (Hopkins et al., 2009) and determined using a published spreadsheet (xPostOnlyCrossover.xls) available online (Hopkins, 2017).

Relationships between athletes’ sleep duration and each outcome measure (mucosal measurements and self-report responses) were assessed using linear mixed models. In these models, sleep duration (minutes) was included as the predictor variable (fixed effect), and separately, mucosal and self-report responses were included as the outcome measure. Using a random intercept and slope design, athlete identification and the corresponding outcome variable were included as random effects. The relationship between sleep duration and the outcome variables were standardized by multiplying the final model slope by 2 × the within-subject standard deviation that were obtained using a mixed model reliability analysis with a random effect for athlete identification (Higham et al., 2014). This method results in the expected change in the outcome measure from a typically low (-1 SD) to a typically high value (+1 SD) (Hopkins et al., 2009). This effect (expressed as a SD) was then converted to an ES using the between-subject standard deviation (obtained from the mixed model reliability analysis), that were categorized using the ES magnitude thresholds as described previously and were also interpreted using the magnitude-based inference approach as stated above. These analyses were performed using customized R Studio statistical software (V 1.1.453), and packages including lme4, lmerTest and emmeans were used.

## Results

**FIGURE 3:**
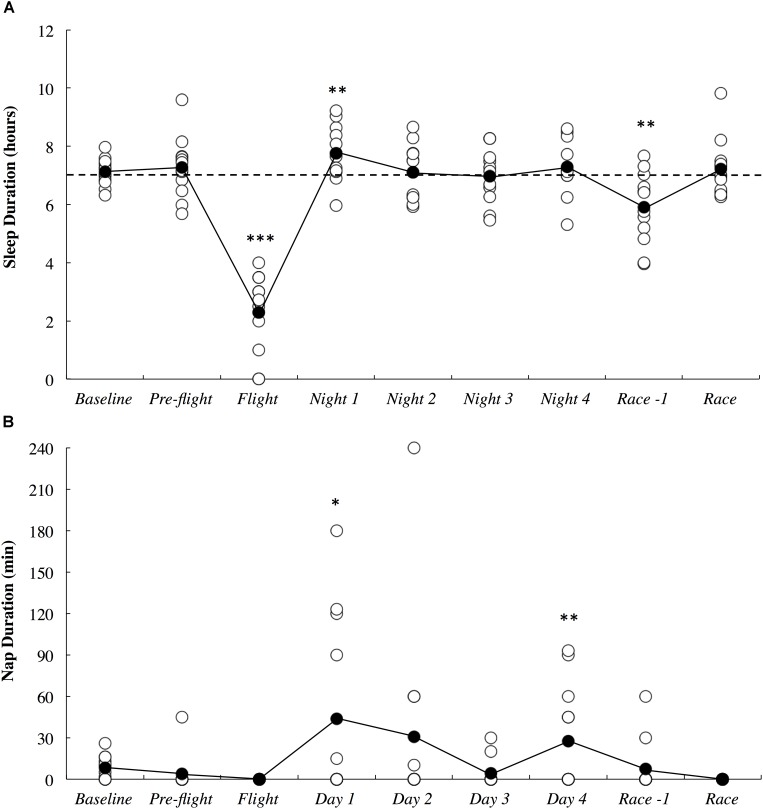
Objective sleep duration **(A)** and subjective nap duration **(B)** across the study period. White circles represent individual responses and black circles represent the mean. Substantial differences compared to baseline are denoted as ^∗^*likely*, ^∗∗^*very likely* and ^∗∗∗^*most likely*. The dotted line represents the minimum sleep duration recommended for adults.

**FIGURE 4:**
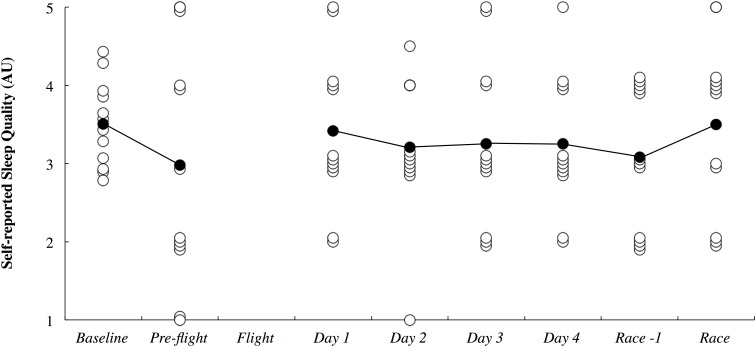
Subjective sleep quality presented in arbitrary units (AU) across the study period. White circles represent individual responses and black circles represent the mean. No sleep quality data was obtained during the flight.

The objective sleep duration and self-reported nap duration are illustrated in Figure 3. When compared to baseline, sleep duration was *most likely* lower during the international flight (3.06; ±1.26) and *very likely* higher on Night 1 (1.15; ±0.92). Sleep duration returned to baseline thereafter until it was *very likely* lower on the night prior to competition (1.18; ±0.93). Compared to baseline, nap duration was *likely* higher on day 1 (3.90; ±3.70) and *very likely* higher on day 4 (2.13; ±1.77). Self-reported sleep quality is illustrated in Figure 4. There were no changes in subjective sleep quality or objective sleep efficiency compared to baseline. No sleep occurred during either of the short domestic flights.

**FIGURE 5:**
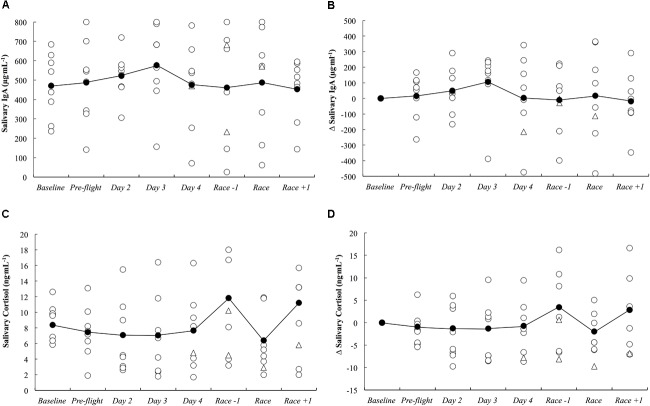
Salivary IgA concentration as absolute **(A)** and delta **(B)** values, and salivary cortisol concentration as absolute **(C)** and delta **(D)** values across the study period. White circles represent individual responses, white triangles represent individual responses when they reported illness symptoms and black circles represent the mean.

The absolute and delta sIgA and sCort concentrations across the study period are illustrated in Figure 5. There were no substantial differences in sIgA and sCort responses compared to baseline on any day throughout the study period.

There were three athletes (25%) who developed symptoms of illness 3–5 days after arrival in the new destination. Athlete A developed ‘severe’ upper respiratory symptoms that persisted for 5 days (including race day). Athlete B developed ‘minimal’ upper respiratory symptoms that persisted for 1 day. Athlete C developed ‘minimal’ symptoms of chest infection and ‘moderate’ symptoms of headache which both persisted for 3 days.

**FIGURE 6:**
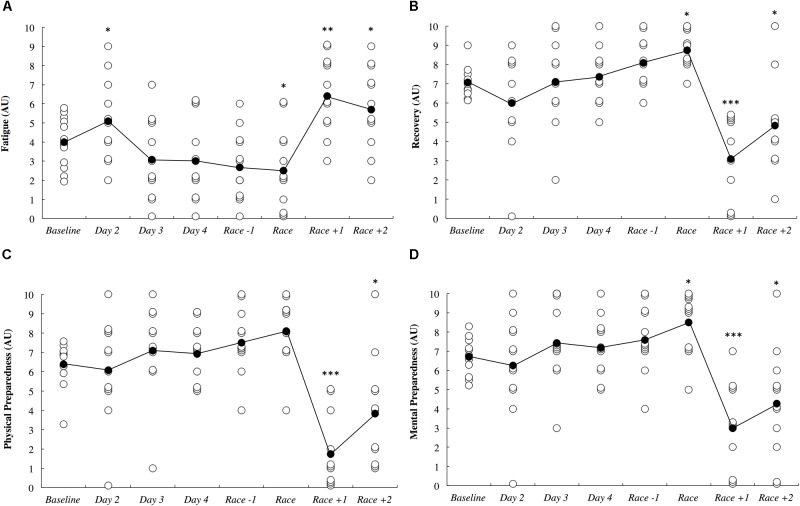
Self-reported fatigue **(A)**, recovery **(B)**, physical preparedness **(C)**, and mental preparedness **(D)** across the study period in arbitrary units (AU). White circles represent individual responses and black circles represent the mean. Substantial differences compared to baseline are denoted as ^∗^*likely*, ^∗∗^*very likely* and ^∗∗∗^*most likely*. No self-report data was collected on day 1.

Subjective measures of fatigue, recovery and preparedness are illustrated in Figure 6. All comparisons are made compared to baseline. Fatigue ratings were *likely* higher upon waking after the first night in the new destination (0.54; ±0.41), *likely* lower on the day of the race (0.78; ±1.27), *very likely* higher on the day after the race (1.58; ±0.96) and *likely* higher 2 days after the race (0.83; ±1.13). Recovery ratings were *likely* higher on the day of the race (1.67; ±2.94), were *most likely* lower the day after the race (2.21; ±0.81) and were *likely* lower 2 days after the race (1.14; ±1.00). Physical preparedness was *most likely* lower the day after the race (2.64; ±0.90) and was *likely* lower 2 days after the race (1.09; ±0.92). Mental preparedness was *likely* higher on the day of the race (1.25; ±1.89), *most likely* lower on the day after the race (1.91; ±0.83) and was *likely* lower 2 days after the race (1.03; ±0.88).

There were meaningful associations between sleep duration and various self-reported measures. Specifically, sleep had a *very likely* association with both fatigue (0.92; ±0.55) and perceived sleep quality (0.84; ±0.64). There was also a *likely* negative association between sleep duration and recovery (-1.04; ±1.21). Associations between sleep duration and sIgA and sCort were unclear.

**FIGURE 7:**
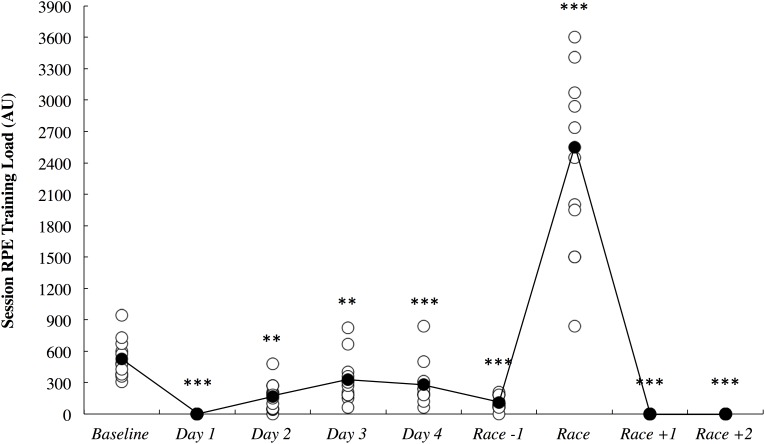
Session rating of perceived exertion (RPE) training load across the study period in arbitrary units (AU). White circles represent individual responses and black circles represent the mean. Substantial differences compared to baseline are denoted as ^∗∗^*very likely* and ^∗∗∗^*most likely*.

The session RPE training load data is illustrated in Figure 7. All comparisons are made to baseline measurements. All of the athletes had a rest day on day 1, and as such, training load was *most likely* lower on this day (2.65; ±0.48). Training load was *very likely* lower on day 2 (5.30; ±3.34) and day 3 (1.78; ±1.07) until it was *most likely* lower on day 4 (2.22; ±1.03) and the day before the race (5.98; ±2.76). Training load was *most likely* higher on the day of the race (4.32; ±0.80) and *most likely* lower on both days following the race when no training was completed (2.65; ±0.48).

Total Hawaii Ironman 70.3 race duration for male (range h:min: 4:59–6:41) and female (7:42–8:30) athletes show some variance. These durations do not securely reflect the training status of the athletes, due to the extreme heat and arduous terrain of this event.

## Discussion

The current study demonstrated that long-haul northeast travel from a cool to a hot environment had *most likely* and *likely* negative influences on sleep and self-reported fatigue, respectively, in masters level endurance athletes. Sleep duration was *most likely* decreased on the overnight flight, and to compensate, nap duration was *likely* increased on the first day, and sleep duration was *very* *likely* increased on the first night after arrival (Figure 3). Upon waking after the first night, the athletes reported *likely* greater fatigue (Figure 6A). Three athletes (25%) developed symptoms of illness 3–5 days after arrival at the destination, but there were no changes in mucosal markers of immunity or stress throughout the study period (Figure 5). As such, hypothesizes ‘i’ and ‘iii’ are accepted, and hypothesis ‘ii’ is rejected. On the day of competition (Hawaii Ironman 70.3), fatigue, recovery and mental preparedness were *likely* improved (Figure 6) corresponding with the tapering training program (Figure 7), but sleep duration was *very likely* decreased on the night prior to the competition. Overall, these findings are important and useful for practitioners when planning competition that involves international long-haul travel. In particular, interventions are may assist in reducing sleep disruption during travel and the night prior to competition, together with minimizing the risk of illness.

Sleep duration was dramatically lower on the overnight international flight compared to a normal night of sleep when the athletes were in their own homes (Figure 3A). This observation may have been caused by the uncomfortable upright seating/sleeping position (Roach et al., 2018), light, noise and the unusual environment and routine experienced on the flight. The *very likely* increased sleep duration observed on the first night after arrival is a common occurrence due to a greater homeostatic sleep pressure as a result of reduced sleep duration on the flight (Fowler et al., 2015; Fullagar et al., 2016)]. On average, however, more sleep duration was lost during the flight (4.8 h) compared to what was regained through daytime napping (0.7 h) and sleeping (0.7 h) on the first day/night after arrival. These athletes may have had difficulty catching up on the sleep lost on this first night due to: (i) additional daytime napping interrupting evening sleep; (ii) additional evening socializing associated with staying in an overseas resort with friends and family; (iii) early awakening due to the change in time-zone (-22 h) and; (iv) scheduled morning training at 07:00. Hence, the athletes may have found it difficult to get to sleep any earlier than normal in the new environment, while still having morning training demands (albeit slightly later than usual) on each day after arrival. Relative to the American National Sleep Foundation, several of the athletes slept less than the recommended minimum duration of 7 h for adults (see Figure 3A) both at baseline and throughout the study period (Hirshkowitz et al., 2015). For these athletes, sleep education and sleep hygiene interventions may be beneficial before traveling, while the major challenge for athletes already sleeping 7 h and above would be compensating for sleep lost upon arrival.

The athletes reported *likely* increased fatigue on the morning after the first night in the new destination, possibly due to the lack of sleep on the flight, combined with the difficulty of compensating for lost sleep on the first night in the new environment. Living in the hotter environment may have also contributed to the additional fatigue, as a large amount of time was spent outside in the heat (away from air conditioning). Both sleep duration, and self-reported fatigue had returned to baseline by the second night and third morning after arrival, respectively. More generally, high levels of fatigue and low levels of recovery were associated with increased sleep duration across the study period, as identified via linear mixed models. Therefore, the first 48 h after long-haul travel is perhaps a key time to optimize sleep hygiene practices in athletes traveling for competition. Sleep could also be positively influenced by employing napping to further compensate for the air-travel induced decrements in total sleep duration. While some of the athletes napped on the day of arrival (5/12) and the day after arrival (4/12), this strategy could have been beneficial for all athletes. Indeed, evidence suggests that naps timed appropriately after previous exercise and taken later in the day (13:00–15:00) may positively influence an athletes willingness to engage in further physical and mental efforts (Bonnar et al., 2018). Further, findings regarding professional rugby league athletes demonstrated napping did not impede night time sleep, rather increasing total sleep quality and quantity (Thornton et al., 2017). Other acute sleep hygiene strategies (e.g., appropriate lighting, electronic device availability, cool room temperature, ear plugs, eye masks, etc.) have had varying effects on sleep quality and duration in athletes (Bonnar et al., 2018) and could be employed on an individual basis as needed.

While others have demonstrated significant associations between a habitual sleep duration of <6 h and an increased risk of the common cold (Prather et al., 2015), we observed no changes in mucosal markers of sIgA or sCort (Figure 5) on day 2 onward following the overnight travel (sleep duration on the flight was <4 h in all athletes). Indeed, associations between sleep duration and sIgA and sCort were unclear within the present data. However, the mucosal data may be confounded by the much lower training loads completed upon arrival compared to baseline (Figure 7), as the intense and prolonged training completed at baseline is known to reduce sIgA (Mackinnon and Hooper, 1994) and hence, sIgA may be naturally increased under the lighter training loads after the travel, making it difficult to identify lower sIgA as a risk factor for URTI at this time. Further, saliva samples could not be obtained at the usual time of day on day 1 due to logistical constraints of being on the airplane/in the terminal, meaning it is unknown if the mucosal responses were altered upon waking/immediately after the international flight. Nevertheless, three athletes (25%) developed symptoms of illness (URTI or chest infection), including one athlete for whom severe symptoms persisted for 5 days (including the day of competition). This illness frequency distribution is similar to what has been reported previously in those who slept an average of 5–6 h (29%) and 6–7 h (23%) across a 7-day period (Prather et al., 2015). The *likely* – *very likely* differences in sleep and fatigue identified in this manuscript all occurred in the first 2 days after arrival, before any symptoms of illness were reported. Therefore, the illnesses that were self-reported were not impacting on the major outcomes of this investigation. However, the illnesses experienced after this time would have affected the self-report measures, but evidently, not by enough to affect the outcomes of the study (i.e., there were no other *likely*, *very likely* or *most likely* changes compared to baseline in the negative direction), and as such the participants that reported illness were not removed from analysis of the self-report data.

Fatigue, recovery and mental preparedness were *likely* improved compared to baseline on the morning of competition (Figure 6), which may be due to the tapering training program (Figure 7) and other individual race day preparation strategies that may have been used by the athletes (e.g., pre-race routines). It is not surprising that all of these measures, as well as physical preparedness, were *very likely* deteriorated in the days following the 5- to 8-h triathlon. It should be noted that sleep duration was also *very likely* lower on the night prior to competition (Figure 3A), perhaps a result of the required early wake up time on this day (3:30 AM), combined with feelings of anxiety (Juliff et al., 2015). Hence, the sleep hygiene practices discussed above may also be beneficial for athletes to implement on the night prior to competition. There is evidence in the current study that some of the athletes increased their nap duration in the days prior to competition (Figure 3B), perhaps in preparation for a lack of sleep on the night prior to the race.

It is important to identify the limitations of the current study. Firstly, the baseline measures were taken during normal training weeks, when some fatigue was present. Other studies have established baseline sleep and mucosal measurements during rest weeks (Coad et al., 2016), although this was not possible in the current design and with triathletes, who generally train year round. Saliva samples were analyzed with a portable point of care device, which had lower (but acceptable) reliability compared to laboratory techniques (Coad et al., 2015; Fisher et al., 2015). Further, saliva and self-report data were not obtained upon waking from the overnight flight due to logistical constraints, which potentially coincided with the time when immunity, stress and fatigue were challenged the most. Two different airlines were used by the athletes when flying from Sydney to Honolulu, which created a small variance in the stopover duration at Sydney airport, however, the flight duration was the same (and hence there were very similar routines on the flights themselves in terms of meals, light exposure and physical activity). It should also be noted that self-reported symptoms of illness do not necessarily reflect illness as diagnosed by a physician or detected by the presence of a pathogen (Gleeson, 2007). Finally, the travel fatigue and the hotter environment could not be isolated in the current study, but they represent two combined contextual factors that athletes may face when traveling to a hot environment for competition.

## Conclusion

Long-haul northeast travel from a cool to a hot environment had *most likely* and *likely* negative influences on sleep and self-reported fatigue, respectively, but these alterations had returned to pre-departure baseline within 48 h after arrival. This travel appeared not to challenge immunity or physiological stress, when quantified using mucosal markers of sIgA and cortisol. Nevertheless, three athletes (25%) did self-report symptoms of illness 3–5 days after arrival. Endurance athletes undertaking similar journeys may benefit from optimizing sleep hygiene, especially on the first 2 days after arrival, or until sleep duration and fatigue levels return to normal.

## Author Contributions

CS collected the data and wrote the manuscript. CS, HT and CE analyzed the data. All authors developed the research question and study design. All authors provided critical feedback and revisions to the manuscript drafts.

## Conflict of Interest Statement

The authors declare that the research was conducted in the absence of any commercial or financial relationships that could be construed as a potential conflict of interest.
